# Balancing Ethics and Culture: A Scoping Review of Ethico-Cultural and Implementation Challenges of the Individual-Based Consent Model in African Research

**DOI:** 10.1177/15562646241237669

**Published:** 2024-03-18

**Authors:** Richard Appiah, Giuseppe Raviola, Benedict Weobong

**Affiliations:** 1 Department of Psychology, Faculty of Health and Life Sciences, 5995 Northumbria University, Newcastle upon Tyne, UK; 2 Center for African Studies, Harvard University, Cambridge, MA, USA; 3 College of Health Sciences, 58835 University of Ghana, Accra, Ghana; 4 Department of Psychology, University of Johannesburg, Johannesburg, South Africa; 5 Department of Global Health and Social Medicine, 1811 Harvard Medical School, Boston, MA, USA; 6 Department of Psychiatry, Massachusetts General Hospital, Boston, MA, USA; 7 School of Global Health, Faculty of Health, York University, Toronto, ON, Canada

**Keywords:** informed consent, collectivistic African society, ethical and cultural issues, Africa

## Abstract

**Objective:** This review explores the ethico-cultural and implementation challenges associated with the individual-based informed consent (IC) model in the relatively collectivistic African context and examines suggested approaches to manage them. **Methods:** We searched four databases for peer-reviewed studies published in English between 2000 to 2023 that examined the ethico-cultural and implementation challenges associated with the IC model in Africa. **Results:** Findings suggest that the individual-based IC model largely misaligns with certain African social values and ethos and subverts the authority and functions of community gatekeepers. Three recommendations were proffered to manage these challenges, that researchers should: adopt a multi-step approach to IC, conduct a rapid ethical assessment, and generate an African-centered IC model. **Conclusions:** A pluriversal, context-specific, multi-step IC model that critically harmonizes the cultural values of the local population and the general principles of IC can minimize ethics dumping, safeguard the integrity of the research process, and promote respectful engagement.

## Introduction

The past decade has witnessed a remarkable increase in biomedical and social science research, with collaborations spanning across continents (Coccia & Bozeman, 2016; Lee & Haupt, 2021). While this cross-cultural exchange of knowledge has enriched scientific advancements, it has also raised critical questions about the applicability and adaptability of established research methodologies, particularly in the Global South (Haelewaters et al., 2021; Lunn, 2014). In their well-intentioned quest to adhere to global standards, researchers often inadvertently employ a standardized approach that may overlook the intricate sociocultural dynamics, potentially undermining the cultural norms, values, practices, and integrity of research participants in the majority world, particularly within certain African contexts (Appiah, 2022; Wilson Fadiji et al., 2022). Central to the research ethical paradigm is the aspiration to safeguard individual autonomy, ensuring that participants are not only cognizant of the research objectives but also willingly engage in the process (Beauchamp & Childress, 2019). That research and medical procedures should essentially benefit or promote the good of other persons, and that research should be conducted in fairness for individuals, groups, and communities, are widely acknowledged concepts with unified interpretations across contexts and cultures (Anabo et al., 2019; Miracle, 2016). However, the conceptualization of individual autonomy, rooted in a Western individualistic ethos, may be discordant with the prevailing communal ethos found in non-Western societies, such as Africa and Asia, where social cohesion, familial interconnectedness, and communal decision-making often overshadow individual autonomy (Appiah, 2021; Visagie et al., 2019).

Although the collective contributions of the four fundamental principles of (research) ethics (i.e., autonomy, non-maleficence, beneficence, and justice) in advocating and propagating ethical research and practice have been widely acknowledged, the principle of ‘respect for autonomy’ has been criticized by scholars and ethicists for its overemphasis on individual autonomy (Metz, 2017), and for its failure in recognizing the variations in social orientations and the conception of personhood across cultures (Behrens, 2018). For instance, Behrens (2018) argued that ‘respect for autonomy’ is insufficiently narrow as a basic principle of bioethics given its individualistic orientation, and that it ignores the important fact that individuals are embedded within communities. Instead, Behrens (2018) re-emphasized the universality of the principle of ‘respect for persons’ (as originally postulated in the Belmont Report) because of its propensity to recognize the decisional authority of individuals and their relationality with others. Other scholars and bioethicists have shared similar views, arguing that the shift of the principle from ‘respect for persons’ to ‘respect for autonomy’, engineered by Beauchamp and Childress, reduces respect to a mere protection of persons from ‘non-interference’ (Lysaught, 2004; Wispe, 2022).

Traditionally, the standard informed consent (IC) adopts an individual-based consent model, where norms of decision-making emphasize respect for individual autonomy (Azétsop & Rennie, 2010; Nishimura et al., 2013). The question of whether all principles and ethical guidelines (e.g., the autonomy-focused IC model) are universally applicable to all research contexts has been raised by researchers working in the relatively collectivistic context of Africa (Appiah, 2020, 2021; Molyneux et al., 2005; Tekola et al., 2009; Vreeman et al., 2012a). Largely, collectivist cultures foster a sense of the self that emphasizes interpersonal harmony rather than individual private engagement (Gavi et al., 2022; Gyekye, 2011; Mpofu, 1994), with an individual's self-identity often conceived in terms of their connectedness with other members of the family or society. In spite of their close affinity to family and clan, individuals in collectivist societies are responsible for taking their personal life decisions (Molefe, 2018), and society, for the most part, neither determine one's actions or choices nor dictates specific rational life plan for everyone (Ikuenobe, 2018). Collectivists, however, are expected to take others into account in their decision-making and to contribute to advancing the goals of the group (Gavi et al., 2022; Mpofu, 1994), engendering an elevated notion of ‘duty to others’. This contrasts with the notion of ‘individual rights’ in Western social orientation, where the emphasis is placed on personal independence, self-determination, and privacy (Behrens, 2018).

While much of the social organizations of Asia and South America have been described as collectivistically socially oriented rather than individualistic (Ikuenobe, 2018; Markus & Kitayama, 1991), the anthropological literature describes African societies as among the most collectivist in their construction and view of the self (Eaton & Louw, 2000; Gyekye, 1996; Triandis, 1989). In most of sub-Saharan Africa, individuals belong to an extended family system, are loyal to their clans, and may use teknonyms as part of their social affiliation and identities (Gyekye, 2014; Mpofu, 1994; Nwoye, 2017). In the more rural, close-knitted, relatively collectivistic context of sub-Saharan Africa, for instance, a person's failure to show concern or participate in the resolution of a misfortune of a member of the family or community could be construed as perfunctory and unceremonious, which is frowned upon (Gyekye, 2011). A person's time and resources spent in helping others to resolve their problems are regarded as the individual's contribution to advance the victim's freedom – as this contribution essentially alleviates the anxiety and stress associated with the problem. The collectivism notion of the African people is ubiquitously explicated by Kwame Gyekye, a renowned Ghanaian philosopher, that in the Akan and other African moral systems, an individual can be a *human being* without being a *person*, thus distinguishing between the concept of a human being and the concept of moral personhood (Gyekye, 2011). Gyekye further argued that, people, functioning as human beings, are naturally oriented toward other persons, hence are social beings who require social ethics rather than ethics of individualism, explaining that Aristotle's dictum, *politikon*, is better translated as ‘social’ rather than ‘political’ (Gyekye, 2011).

Nevertheless, similar to many regions of the world, the social organization of many African societies largely co-exist as collectivist and individualist orientations, with a collectivist orientation more pronounced in the culturally-laden rural and peri-urban settings, in contrast to the characteristics of individualist orientations that are observed in the urban and industrialized settings (Ikuenobe, 2018; Phillips & Wong, 2016). In the rural, relatively communal settings of Ghana, for instance, despite acknowledging their individual identities, residents express a high sense of social interconnectivity, with the decision-making process of prospective research participants and the processes to obtain IC taking on greater complexity (Appiah, 2021). Yet, the question of whether and to what degree cultural norms and practices (e.g., gatekeepers’ involvement in the research and decision-making processes) violate the individual rights of prospective participants and/or its effect on the integrity of the research process is the cause of considerable debate (Honan et al., 2013; Liamputtong, 2008; Marshall & Batten, 2003; Obijiofor et al., 2018; Ojeda et al., 2011).

At the core of research ethics lies the objective of safeguarding individual autonomy and welfare. However, the very concept of autonomy can take on varied meanings when transposed onto societies with deeply rooted communal identities (Visagie et al., 2019). The principles of individual autonomy that guide the IC process may diverge from the priorities and norms prevalent in collectivistic societies like Africa (Appiah, 2020, 2021; Behrens, 2018). In many African and Asian cultures, community interests often take precedence over individual desires (Gyekye, 1996, 2014; Mpofu, 1994; Nwoye, 2017), thereby posing a complex challenge to the conventional IC model. This incongruence between Western ethical norms and the ethico-cultural underpinnings of Africa (and much of the Global South), necessitates a comprehensive investigation into the specific challenges that arise when applying the individual-based IC model within African contexts. Although a growing body of literature across Africa suggests that the standard, individual autonomy-oriented IC model presents ethico-cultural and implementation challenges when applied in the relatively collectivistic African cultural context (Marshall et al., 2014; Visagie et al., 2019), the extent and implications of these challenges on the integrity of the research process are not known. Research guidelines also generally take a more global perspective that typically posit a universalism that obscures their profound alignment with the cultural norms of ‘Western’ thought, thus raising critical concerns about their cultural-fitness and utility in non-Western cultural settings (Schroeder et al., 2019).

Drawing on empirical studies and theoretical models of bioethics, Barugahare (2018) provides some methodological insights for bioethics in Africa, pertinent to addressing challenges associated with implementing individual-based IC frameworks in relatively collectivistic contexts of Africa. Firstly, Barugahare contends that while embracing African perspectives, it is crucial for researchers and practitioners to maintain a balanced approach that respects diverse moral viewpoints (Barugahare, 2018). This inclusive stance ensures culturally sensitive practices without compromising universal ethical principles, which are also vital for navigating the ethico-cultural and practical challenges reported to confront the administration of individual-based IC frameworks in certain contexts of Africa. Secondly, Barugahare (2018) suggests that (bio)ethical principles should be assessed for their inherent value rather than their origin. This recommendation urges researchers and practitioners to conduct an impartial analysis to identify ethical frameworks that align with African values and effectively address contemporary health (and social) challenges, facilitating the development of robust research and clinical practices while transcending cultural biases. Barugahare further proposed that rather than dismissing Western (bio)ethics, efforts should focus on integrating global standards with African moral insights. This perspective resonates with the extant literature that suggests that adapting existing principles ensures ethical standards are upheld while respecting cultural norms. Lastly, Barugahare (2018) argued that critical thinking abilities are essential for navigating complex ethical issues in research and practice, and challenges researchers to foster intellectual rigor in order to address challenges with (bio)ethical frameworks, ensuring decisions are grounded in reasoned analysis rather than cultural biases.

Although recent efforts have generated toolkits to support researchers to navigate research ethical conflicts (Reid et al., 2021), they do not provide detailed descriptions of the ethical, cultural, and practical challenges akin to specific cultural contexts. Identifying and characterizing the challenges associated with the IC and research processes, particularly in low- and middle-income countries (LMICs) could enhance our understanding of the research trajectory and challenges in specific contexts and inform the development (or adaptation) of context-tailored IC approaches. Insights from such efforts could also facilitate the conduct of ethical research in underserved communities and thereby minimize ethics dumping – the export of unethical research practices from high-income to lower-income settings (Schroeder et al., 2018, 2021). This review explored scholarly literature describing the ethico-cultural and implementation challenges associated with the universal IC framework in Africa, in order to identify the nature and extent of these challenges. The study also identifies strategies suggested in the literature to manage the challenges more appropriately. This review was guided by the questions: a) What ethical, cultural, and implementation challenges are associated with the IC model when applied in research in the African context?, and b) What are the suggested recommendations to manage these challenges more appropriately?

## Methods

We followed the scoping review methodological framework suggested by Arksey and O’Malley (Arksey & O'Malley, 2005), and additionally relied on the (Preferred Reporting Items for Systematic reviews and Meta-Analyses extension for Scoping Reviews (PRISMA-ScR) guidelines (Tricco et al., 2018) to inform the development of the search, selection, synthesis, charting, and interpretation of the results of this review. We systematically searched across four databases, including PubMed, PsycINFO, Web of Science, and Scopus for literature examining or describing the ethico-cultural and implementation challenges associated with the IC model or procedures in Africa. Our research team consists of individuals with expertise in public health, positive psychology, global mental health, community-based participatory research, and research methods.

The search strategies were developed by an experienced librarian, and further refined in consensus with the research team. A combination of search terms relating to topical (“Consent” OR “Informed consent model” OR “Informed consent process” OR “Consent procedures” OR “Consent practices”) AND disciplinary (“Biomedical research” OR “Medical research” OR “Social science research”) AND conceptual (“Ethical challenges” OR “Ethical issues” OR “Ethical considerations”) AND location (“Africa” OR “African countries” OR “sub-Saharan Africa” OR “Algeria” OR “Angola” OR “Benin” OR “Botswana” OR “Burkina Faso” OR “Burundi” OR “Cabo Verde” OR “Cameroon” OR “Central African Republic” OR “Chad” OR “Comoros” OR “Congo” OR “Djibouti” OR “Egypt” OR “Equatorial Guinea” OR “Eritrea” OR “Eswatini” OR “Ethiopia” OR “Gabon” OR “Gambia” OR “Ghana” OR “Guinea” OR “Guinea-Bissau” OR “Ivory Coast” OR “Kenya” OR “Lesotho” OR “Liberia” OR “Libya” OR “Madagascar” OR “Malawi” OR “Mali” OR “Mauritania” OR “Mauritius” OR “Morocco” OR “Mozambique” OR “Namibia” OR “Niger” OR “Nigeria” OR “Rwanda” OR “Sao Tome and Principe” OR “Senegal” OR “Seychelles” OR “Sierra Leone” OR “Somalia” OR “South Africa” OR “South Sudan” OR “Sudan” OR “Tanzania” OR “Togo” OR “Tunisia” OR “Uganda” OR “Zambia” OR “Zimbabwe”) were applied. The final search string is included as Supplementary file (eTable 1). Studies were eligible for inclusion if they were peer-reviewed biomedical, medical, or social science studies, set out to examine or describe the ethico-cultural and implementation challenges associated with the IC model or process in Africa, and were published in English between 2000 to 2023. The search was not limited to any specific type of study design. Gray literature was excluded. We broadly operationalized ethico-cultural and implementation challenges to include participants’ conception and expression of autonomy and communitarianism (i.e., how the emphasis on individual autonomy in the IC model aligns with or conflicts with traditional communitarian values prevalent in African cultures); cultural beliefs and practices (i.e., how cultural beliefs, rituals, and practices intersect with the Western concept of IC and how they influence decision-making); community engagement (i.e., the strategies for engaging communities in the development and/or implementation of IC processes that are culturally appropriate and respectful); and family and community decision-making (i.e., the role of family members and community leaders in making decisions about research participation and how this dynamic interacts with the individual-based consent model). The electronic search was conducted in October 2022 and updated in July 2023. A total of 82 articles were retrieved from the databases. All citations were imported into EndNote to remove duplicates using the automatic deduplication function, reducing the number to 78. Fourteen articles were excluded after the initial title and abstract screening. We performed full text review of 64 articles and excluded 29 articles after the inclusion criteria were applied. 

**Figure 1.**
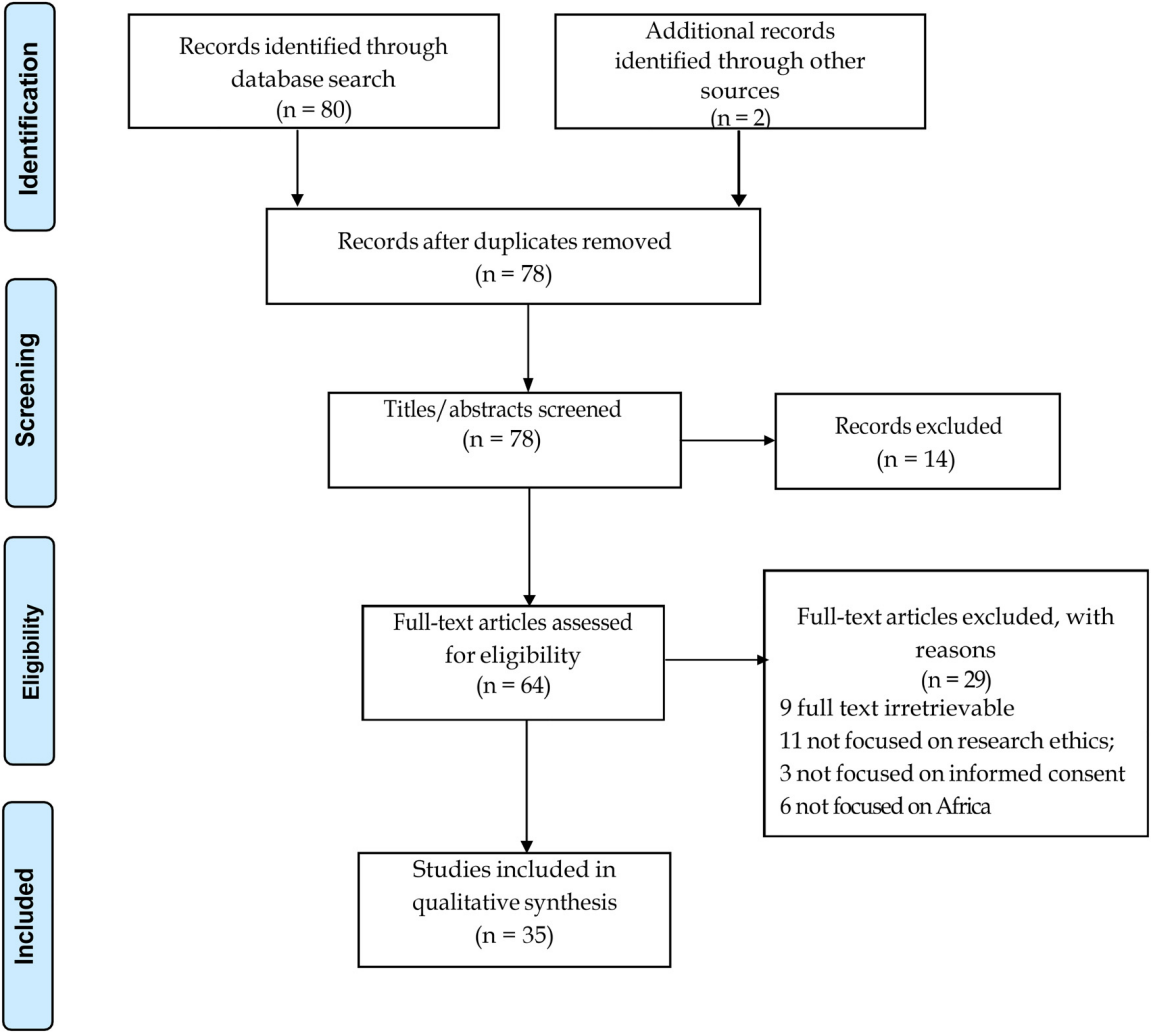
PRISMA flowchart.

We used descriptive numerical summary and qualitative thematic analysis to analyze the extracted data, which were then presented in Tables and Figures. Following Clarke and colleagues’ principles of thematic analysis (Clarke et al., 2015), the first author and an expert reviewer independently read the extractions severally to familiarize themselves, coded the data, and searched for patterns of emerging themes by comparing the data in order to group similar concepts into categories. Findings were compared and differences reconciled through consensual discussions.

**Table 1.** Summary of Articles.

	Publication details	Aim	Setting	Methodological approach	Findings
1	Abay et al. (2016). Rapid ethical assessment on informed consent content and procedure in Hintalo-Wajirat, northern Ethiopia: A qualitative study.	To explore the effects of social, cultural, and religious factors during informed consent process on a proposed HPV-serotype prevalence study.	Ethiopia	A community-based qualitative study	The rapid ethical assessment can potentially reveal several socio-cultural issues relevant to the pending study, and provide valuable insights on how these issues can better be handled, such as participants’ awareness about the research, their rights in research, and whether they prefer verbal consent to written consent.
2	Abdel-Messih et al. (2008). Developing cultural competence and overcoming ethical challenges in the informed consent process: An experience from Egypt.	To discuss some ethico-cultural issues and challenges confronting researchers in Egypt	Egypt	Commentary	The universal research ethical standards requiring participants to append signature or thumbprint the informed consent form may be challenging for researchers working with culturally-diverse populations with low literacy.
3	Addissie et al. (2014). A mixed-methods study on perceptions towards use of rapid ethical assessment to improve informed consent processes for health research in a low-income setting.	To assess the perceived relevance of introducing rapid ethical assessment as a mainstream tool in Ethiopia.	Ethiopia	Mixed-methods	The majority of study participants (95.4%) believed that the informed consent process should be contextualized to the study setting, with a considerable number (39.4%) also thinking that rapid ethical assessment would be an appropriate approach to improve the perceived problems with the consent process.
4	Addissie et al. (2016). Cluster randomized trial assessing the effects of rapid ethical assessment on informed consent comprehension in a low-resource setting.	To assess the effects of rapid ethical assessment on comprehension, retention and quality of the informed consent process.	Ethiopia	A cluster randomized trial	A large proportion of participants in the intervention group, but not the control group, showed a higher level of comprehension of the informed consent process and were satisfied with the overall consent process, suggesting that concerted efforts to tailor the consent framework and processes to the local setting are important and laudatory.
5	Akpa-Inyang and Chima (2021). South African traditional values and beliefs regarding informed consent and limitations of the principle of respect for autonomy in African communities: a cross-cultural qualitative study.	This study explored African biomedical researchers’ perspectives regarding informed consent and potential limitations to the principle of respect for autonomy in African communities.	South Africa	Qualitative study	There are theoretical and practical challenges with applying the standardized, libertarian rights-based autonomy informed consent framework in the more collectivistically-socially oriented African context.
6	Appiah (2021). Gurus and griots: Revisiting the research informed consent process in rural African contexts.	To discuss some theoretical, ethico-cultural, and methodological challenges associated with applying the universal, Western individualistic cultural value-laden IC process in sub-Saharan Africa.	Ghana	Commentary	Rather than adopt a universal one-size-fits-all informed consent approach, researchers working in the rural, highly collectivistic, low literate, socioeconomically disadvantaged settings of sub-Saharan Africa should deeply consider the roles and influence of cultural values and traditional practices on the informed consent and the research process.
7	Appiah (2020). Community-based participatory research in rural African contexts: Ethico-cultural considerations and lessons from Ghana	To examine a range of ethico-cultural issues associated with community-based group intervention research in rural remote settings of Ghana.	Ghana	Commentary	Researchers working in the rural, more collective social settings of Ghana may be confronted with a distinct set of cultural and ethical issues. Researcher should work along with participants and gatekeepers (who are often supportive of the research) to draw on sociocultural resources to design diversified informed consent models to facilitate the research process.
8	Araali (2011). Perceptions of research assistants on how their research participants view informed consent and its documentation in Africa	To typify and exemplify the African perspective with regard to procedures for obtaining consent (agreement) and for documenting it.	Democratic Republic of Congo	Mixed methods	Majority of participants felt uneasy, disliked the idea of signing the consent form, were hesitant, or found signing the consent form strange, and that the consent form commits them to the unknown as a means to exploit them.
9	Behrens (2018). A critique of the principle of ‘respect for autonomy’, grounded in African thought.	To discuss how the principle of ‘respect for autonomy’ dominates the field of bioethics, and how it came to triumph over its competitors, ‘respect for persons’ and ‘respect for free power of choice’.	Africa	Commentary	‘Respect for autonomy’ is unsatisfactory as a basic principle of bioethics in African societies because it is grounded in too individualistic a worldview; the principle fails to acknowledge the fundamental importance of understanding persons within the nexus of their communal relationships.
10	Brear (2018). Ethical research practice or undue influence? Symbolic power in community-and individual-level informed consent processes in community-based participatory research in Swaziland.	To apply Bourdieu's theory to interrogate the social relations of informed consent in the “field” of community-based participatory research in a rural Swazi community, and specifically, to investigate how obtaining community-level consent influenced community members’ autonomy to provide individual-level consent.	Eswatini	Qualitative study	Participants recognized the essence of involving community leaders in the informed consent in community-based participatory research process. It was however noted that since community leaders uphold “symbolic power”, their involvement could potentially constrain individual agency and reproduce existing relations of power, but not when the informed consent process is introduced and implemented on the basis of autonomy and rights of research participants.
11	Chukwuneke et al. (2014). Global bioethics and culture in a pluralistic world: How does culture influence bioethics in Africa?	To examine the cultural influence on the principles and practice of bioethics in Africa.	Africa	Narrative review	In most of Africa, individuals are expected to owe allegiance to their family and communal systems, and in certain situations need to involve or consider the well-being of the family or community in their decision-making.
12	Diallo et al. (2005). Community permission for medical research in developing countries.	To describe the process used to obtain community permission at a malaria research site in Mali, and the rationale behind it.	Mali	Commentary	There was the recognition that, like obtaining individual consent, obtaining community permission is a dynamic and important process in the research context. The consultation process was amended in a formal meeting with community leaders to allow consent at both communal and individual levels.
13	Dunin De Skrzynno and Di Maggio (2018). Surgical consent in sub-Saharan Africa: a modern challenge for the humanitarian surgeon.	To recount the authors’ personal experiences of surgical consent in Burundi and to review the literature describing its practice and the specific challenges faced in Sub-Saharan Africa.	Burundi	Commentary	Authors support the World Medical Association's stance on autonomy and decision-making, but recommends that the process for obtaining informed consent should be reviewed and developed to integrate the cultural norms of the local population, given that informed consent is influenced by cultural background, family structure, socioeconomic status, religion and education.
14	Frimpong-Mansoh (2008). Culture and voluntary informed consent in African health care systems	Discusses strategies to apply a context-appropriate, collective decision informed consent model in a highly collective African settings in order to minimize exploitations and ethics dumping.	Africa	Perspectives	Because people in more communal settings have strong social relations with family and community that also significantly influence their behavior and view of reality, it is insufficient and inappropriately narrow to confine medical decisions to individual patients and their self-centered choices: individual rights have limits.
15	Gebresilase et al. (2017). Rapid Ethical Appraisal: A tool to design a contextualized consent process for a genetic study of podoconiosis in Ethiopia.	To employ a rapid ethical assessment tool to assess local barriers to genuine informed consent prior to conducting a genetic study of podoconiosis (non-filarial elephantiasis) in two Zones of Ethiopia.	Ethiopia	Qualitative study	Participants felt comfortable when approached in the presence of trusted community members, and preferred that both verbal and written informed consent processes are administered.
16	Gondwe et al. (2022). Guardians and research staff experiences and views about the consent process in hospital-based paediatric research studies in urban Malawi: A qualitative study.	To explore the views of guardians and researchers about the consent process in Malawi in order to inform the develop appropriate consent guidelines.	Malawi	Qualitative study	Guardians of children participating in paediatric clinical trial and observational studies felt that the role of their spouses was neglected during consenting, while research staff reported that they had problems obtaining consent from guardians when their partners were not present.
17	Jegede (2009). African ethics, health care research and community and individual participation.	To discuss the appropriateness of Western bioethics in the African setting, focusing on the decision-making process regarding participation in health research as a contested boundary in international bioethics discourse.	Africa	Narrative review	Africa largely adopts communal or social autonomy, as opposed to individual autonomy in the West, suggesting that the Western concept of autonomy to research involving human subjects in the African context without adequate consideration for the important role of the community is inappropriate
18	Jeko et al. (2012). Obtaining informed consent in non-Western contexts: Reflections on fieldwork experiences in Zimbabwe.	Authors reflect on their experiences in obtaining informed consent for an educational research study in Zimbabwe using a Western-based ethics protocol.	Zimbabwe	Perspectives	Inasmuch as informed consent protocol valorises the individual autonomy and rights, it is likely to be difficult to implement in African society, who mainly adopts community-oriented culture based on values of togetherness, community feeling and solidarity.
19	Kengne-Ouafo et al. (2014). Perceptions of consent, permission structures and approaches to the community: A rapid ethical assessment performed in North West Cameroon.	To explore ethical issues and challenges associated with approaching communities and gaining informed consent in North West Cameroon, using a rapid ethical assessment approach	Cameroon	Qualitative study	Participants described a centralized permission-giving structure in their communities that they expect researchers to abide by. Nonetheless, there was evidence of some subversion of these structures by the educated individuals.
20	Marshall et al. (2014). Voluntary participation and comprehension of informed consent in a genetic epidemiological study of breast cancer in Nigeria.	To investigate voluntary participation and comprehension of informed consent among women involved in a genetic epidemiological study on breast cancer in Nigeria.	Nigeria	Interviewer- administered surveys	The majority of participants reported being told that their participation was voluntary; that they could withdraw at any time without repercussions. Although no one sought permission from local elders, a considerable number of participants reported asking permission from their husbands before they enrolled in a study.
21	Molyneux et al. (2005). ‘Even if they ask you to stand by a tree all day, you will have to do it (laughter)…!’: community voices on the notion and practice of informed consent for biomedical research in developing countries.	To solicit community members’ perspectives on informed consent procedures, and their suggestions on how to conduct context-appropriate informed consent process.	Kenya	Qualitative study	There was a widespread agreement by community members that chiefs and elders can give approval to researchers to conduct the study in the community, but households and individuals should provide autonomous informed consent.
22	Moodley, K (2002). HIV vaccine trial participation in South Africa–an ethical assessment.	To discuss the ethical incompatibility of applying Western individualistic consent models in HIV vaccine trials in South Africa, and to suggest the concept of ‘‘Ubuntu’’ as an alternative framework for conducting ethical HIV vaccine trials in South Africa.	South Africa	Narrative review	The concepts of informed consent, risk-benefit ratio, and fair treatment of research participants are interpreted differently in traditional, rural African communities, where a moderate form of communitarianism referred to as ‘‘Ubuntu’’ or ‘‘communalism’’ is still prevalent.
23	Moyer et al. (2022). Social complexities of informed consent and assent among young males undergoing voluntary medical male circumcision in Eswatini.	To examine clients’ levels of circumcision-related knowledge following the assent process, as well as how ethical guidelines were enacted in everyday practice in a setting where family dynamics and norms relating to autonomy and consensus make obtaining informed consent complex.	Eswatini	Qualitative study	The individual-based ethical guidelines applied in research in Eswatini are not in harmony with the realities of the Swazi context, where cultural norms and practices related to consent and age-related respect are at odds with presumptions of agency and rational choice of the individual.
24	Nyirenda et al. (2020). Structural coercion in the context of community engagement in global health research conducted in a low resource setting in Africa.	To discuss some of the ethical issues arising from community engagement in a low resource setting.	Malawi	A qualitative ethnographic study	While the involvement of community leaders in research is considered as culturally appropriate in most African settings, authoritarian leadership structures could coerce and undermine individuals’ autonomy to make voluntary decisions about participation.
25	Osamor and Kass (2012). Decision-making and motivation to participate in biomedical research in southwest Nigeria.	To identify the factors motivating people to participate in biomedical research in a traditional Nigerian community, assess the degree to which participants involve others in the decision-making process, and examine issues of autonomy in decision-making for research.	Nigeria	A descriptive cross-sectional study	Three-quarters (78%) of participants discussed the enrollment decision with someone else; 39% reported obtaining permission from a spouse or family member to participate; and half of the female participants reported seeking permission from a spouse before enrolling
26	Princewill et al. (2017). Factors affecting women's autonomous decision making in research participation amongst Yoruba women of Western Nigeria.	To explore the experience and understanding of autonomy by the Yoruba women in relation to research participation.	Nigeria	Qualitative study	Patriarchy, religion and culture are conceived to have negative impact on the autonomy of women with respect to research participation. However, men feel that by making decisions for women, they were protecting them; but the women interpret this ‘protection’ as a way of limiting their autonomy.
27	Ramabu (2020). Whose autonomy is it? Botswana socio-ethical approach to the consenting process.	To reflect on the fieldwork regarding the informed consent process for bioethics research in Botswana communities.	Botswana	Mixed methods	Both policy makers and practitioners in the village and urban areas opted for individual autonomy. However, caregivers from Gaborone city exercised individual autonomy to participate in the study, while participants from the Letlhakeng village requested that permission be obtained from the community leader before they could participate.
28	Rashad et al. (2004). Obtaining informed consent in an Egyptian research study.	To discuss areas where the Arab Muslim interpretation of some ethical principles, especially around the issue of gaining informed consent, differed from that currently accepted in British research ethics.	Egypt	Commentary	In Egyptian society, decision-making usually involves other important members of the family or community such as father, teacher, or employer. Research informed consent should provide opportunities for inputs by these stakeholders.
29	Shaibu, S (2007) Ethical and cultural considerations in informed consent in Botswana	To recount the ethico-cultural challenges confronting researchers when applying the individual-based standardized informed consent model in Botswana.	Botswana	Reflections	Researchers and practitioners must acknowledge the influence of the sociocultural context (e.g., communal norms and cultural values and practices) in the informed consent process, including recognition of the role of the family in the decision-making process, and to an extent, the natural and social environment of the patient and family.
30	Tekola et al. (2009). Tailoring consent to context: Designing an appropriate consent process for a biomedical study in a low-income setting.	To explore factors relating to information and communication during the process of informed consent, and the approach that should be followed for gaining consent.	Ethiopia	Qualitative study	Prospective participants should be approached through, or in the presence of, locally trusted individuals. Community sensitization and group information provision should precede information provision for consent at the individual level.
31	Tindana et al. (2006) The informed consent process in a rural African setting: A case study of the Kassena-Nankana district of northern Ghana	To elicit the views of research participants in the Kassena-Nankana district of Northern Ghana about the informed consent process, specifically about the influence of community leaders and household heads on individuals’ decisions to participate in research.	Ghana	Qualitative study	Chiefs and community elders serve as gatekeepers, from whom the researchers obtain permission before approaching households to recruit individuals into the study. Most participants with low literacy could not read the information in the informed consent and preferred verbal consent instead.
32	Visagie et al. (2019) Informed consent in Africa – Integrating individual and collective autonomy	To provide an overview of some challenges inherent with the informed consent process in rural African communities.	Africa	Narrative review	The research informed consent process in the more collectivistic African context is deeply entrenched in the cultural values and practices of the people, which must be respected. Researchers should consider an African perspective on how to preserve participant autonomy.
33	Vreeman et al. (2012a). Community perspectives on research consent involving vulnerable children in Western Kenya.	To use *mabaraza*, traditional East African community assemblies, to explore how a community in western Kenya viewed participation of children in health research and informed consent and assent processes.	Kenya	Qualitative study	Participants suggested that other caregivers, community leaders, and even community assemblies should be included in the informed consent process.
34	Vreeman et al. (2012b). A qualitative study using traditional community assemblies to investigate community perspectives on informed consent and research participation in western Kenya.	To use *mabaraza*, traditional East African community assemblies, in a qualitative study to understand community perspectives on biomedical research and informed consent within a collaborative, multinational research network in western Kenya.	Kenya	Qualitative study	Participants understood and valued the important role of the informed consent in research, and endorsed an increased role for the community and community leaders in making decisions about research participation, especially in the case of children, through a process of community consent.
35	Zulu et al. (2019). The challenge of community engagement and informed consent in rural Zambia: An example from a pilot study.	To explore the reasons behind the low participation in a school-based pregnancy prevention intervention in rural Zambia, with particular attention to challenges related to the community engagement and informed consent process.	Zambia	Qualitative study	Inadequate use of locally appropriate channels in the dissemination of information and recruitment processes created room for misconstruction and facilitated development of mistrust, undermining the conditions for community engagement and actual informed consent.

## Results

### Study Characteristics

Of the 35 eligible studies, 15 were qualitative studies, 10 were commentaries and perspectives, three adopted mixed methods design, four were narrative reviews, two were surveys, and one cluster randomized trial (Figure 1). The vast majority of the studies were conducted in the context of social science (61%), followed by biomedical (28%), and theoretical (11%) research. In terms of study settings, the majority of the studies were conducted in Ethiopia (5; 14.3%), with an equal number also originating from studies conducted across multiple countries (5; 14.3%). Three studies (8.6%) were each conducted in Ghana, Kenya, and Nigeria; two each (5.7%) in Botswana, Eswatini, Egypt, Malawi, and South Africa; and one each (2.8%) was conducted in Burundi, Cameroon, Mali, Zambia, and Zimbabwe. The majority (86%) of the studies offered some recommendations to guide researchers to navigate the identified challenges. A thematic summary of the ethico-cultural and implementation challenges, together with the suggested approaches to manage them, are discussed below.

### Ethico-Cultural and Implementation Challenges

#### Misalignment with African Social Values and Ethos

Twenty seven studies argued that the autonomy-driven IC model fundamentally presents theoretical and practical challenges when applied in most African settings because it unquestionably conflicts with the collectivistic social orientation and relational notion of personhood expressed in most African communities (Abay et al., 2016; Abdel-Messih et al., 2008; Addissie et al., 2014, 2016; Akpa-Inyang & Chima, 2021; Appiah, 2020, 2021; Araali, 2011; Behrens, 2018; Chukwuneke et al., 2014; Dunin De Skrzynno & Di Maggio, 2018; Frimpong-Mansoh, 2008; Gebresilase et al., 2017; Jegede, 2009; Jeko et al., 2012; Marshall et al., 2014; Molyneux et al., 2005; Moodley, 2002; Moyer et al., 2022; Osamor & Kass, 2012; Princewill et al., 2017; Ramabu, 2020; Rashad et al., 2004; Shaibu, 2007; Tindana et al., 2006; Visagie et al., 2019; Vreeman et al., 2012b; Table 1). These challenges are partly reported to stem from the fact that the principle of autonomy, on many counts, is incongruous with the collectivistic social orientation and the pluralistic conception of personhood in many African communities (Akpa-Inyang & Chima, 2021; Visagie et al., 2019). The autonomy-based IC model places the decisional authority of whether or not to participate in research in an age-appropriate and cognitively capable individual, yet, in many African communities, personhood is largely conceived on social terms (Behrens, 2018; Gyekye, 1996, 2011) and the individual is, for the most part, a part of the whole – who also owes allegiance to the family or community and may involve them in their decision-making (Chukwuneke et al., 2014). A study that examined the degree to which participants in biomedical research in a traditional Nigerian community involved others in the decision-making process reported that three-quarters (78%) of participants discussed the enrollment decision with someone else; whereas 39% reported obtaining permission from a spouse or family member to participate (Osamor & Kass, 2012). In the same study, half of the female participants sought permission from their spouses before enrolling. Another study reports that Africa largely adopts communal or social autonomy, as opposed to individual autonomy, and contended that a strict application of the Western concept of autonomy in research involving human subjects in certain African contexts without careful consideration of the values and beliefs of the research setting, is inappropriately narrow (Jegede, 2009). In a study that compared the IC process between Egypt and the United Kingdom, researchers found that decision-making in the Egyptian society usually involves other important members of the family or community such as father, teacher, or employer (Rashad et al., 2004). The study urged researchers working in the Egyptian context to provide opportunities for inputs by these stakeholders.

Other studies emphasized the discrepancies between individualist social orientations (on which the IC is premised) and the relatively collectivist African sociocultural orientation and viewpoint of personhood (Abdel-Messih et al., 2008; Addissie et al., 2014; Akpa-Inyang & Chima, 2021; Appiah, 2021; Araali, 2011), and the collectivistic rather than the individualistic nature of decision-making in most of Africa (Dunin De Skrzynno & Di Maggio, 2018; Jeko et al., 2012; Moyer et al., 2022; Shaibu, 2007) to explain the cultural inappropriateness and source of the various ethico-cultural challenges associated with the autonomy-driven IC models in parts of Africa.

**Table 2.** Ethico-cultural and Implementation Challenges and Suggested Guidance Related to Informed Consent Identified in the Literature.

	Publication details	Ethico-cultural challenge/s	Implementation challenges	Suggested guidance in the literature
1	Abay et al. (2016). Rapid ethical assessment on informed consent content and procedure in Hintalo-Wajirat, northern Ethiopia: A qualitative study.	Owning to cultural factors and low literacy, participants preferred to provide verbal consent to written consent, often relating the request to append signatures to the consent form to legal accountability.	A considerable number of participants advocated for the inclusion of husbands in the decision-making process, particularly for community-based biomedical and behavioral research.	The rapid ethical assessment revealed important socio-cultural issues that significantly impact the informed consent process. These findings served as invaluable resource in modifying the informed consent information documents to suit the study context.
2	Abdel-Messih et al. (2008). Developing cultural competence and overcoming ethical challenges in the informed consent process: An experience from Egypt.	Stark sociocultural differences between Egyptian and Western societies, which have theoretical and practical implications for autonomous consenting and individual decisional authority.	In some Egyptian communities, appending signatory to a document is a formal and legal procedure, and requesting the signature of a potential participants could signify a lack of trust.	Scholars and research administrators should collaborate with local communities to develop culturally-competent and diversified recruitment and consent procedures that acknowledge and incorporate the cultural, political, and social practices of the local context.
3	Addissie et al. (2014). A mixed-methods study on perceptions towards use of rapid ethical assessment to improve informed consent processes for health research in a low-income setting.	There are vast differences in cultural orientations between Western (which underpins the informed consent framework) and African societies; the design and preparation of consent processes should critically explore and incorporate potential ethical issues relevant to the specific context.	Issues of lack of clarity, inadequate information, language barriers, undue expectations, and power imbalances negatively affect the consent process. There is much emphasis on fulfilling requirements and procedures rather than on genuine concern for the rights and welfare of study subjects; a balance of these two is needed.	Only a fraction of study participants was satisfied with the current standard consent process (with its emphasis on individual autonomy) for research in Ethiopia. The rapid ethical assessment framework is considered relevant by researchers and stakeholders and has the potential to identify and manage challenges with the consent framework.
4	Addissie et al. (2016). Cluster randomized trial assessing the effects of rapid ethical assessment on informed consent comprehension in a low-resource setting.	There are disparities between the requirements of the traditional informed consent framework and the cultural values and practices of the local population, which requires cultural and linguistic modifications to tailor it the context.	None discussed	Researchers should consider to conduct rapid ethical assessments to explore possible cultural issues that can weigh in on the research process, before they launch their main studies.
5	Akpa-Inyang and Chima (2021). South African traditional values and beliefs regarding informed consent and limitations of the principle of respect for autonomy in African communities: a cross-cultural qualitative study.	The standardized informed consent model derives from a Western conception of libertarian rights-based autonomy, contrary to the African collectivist social orientation which is exemplified by the African moral philosophies of *Ubuntu/Botho* and *Ukama,* emphasize communitarianism over individual rights.	A multiplicity of factors, comprising language differences, low educational level, poverty, and cultural beliefs constrained efforts to obtain proper informed consent in South African communities.	There are limitations to applying the principle of respect for autonomy and informed consent in African communities, especially in the context of human biomedical research. Researchers should adopt a more relational approach, such as Ross's prima facie duties, to implement informed consent in African communities.
6	Appiah (2021). Gurus and griots: Revisiting the research informed consent process in rural African contexts.	In communitarian contexts, such as Ghana, personhood is conceived as relational, thus engendering a collective decision-making process, which starkly contrasts with notion of ‘individual rights’ in Western ethics, upon which the informed consent framework is founded.	In some remote settings, participants viewed their participation in a research activity as more or less an altruistic endeavor for the benefit of the community and society, rather than their private engagement — and thus expect that the gatekeepers of the communities (i.e., chief and elders of the community) are consulted for approval before the study commences.	There is need for an African-centred research ethical guidelines for social scientists and researchers working in rural, low-income, collectivistic social settings. The author urged researchers, universities, research institutions, and institutional review boards in Africa to collaborate with local communities and stakeholders to design and implement context-appropriate informed consent framework to prevent ethics dumping and safeguard the integrity of the research process.
7	Appiah (2020). Community-based participatory research in rural African contexts: Ethico-cultural considerations and lessons from Ghana	In the rural, more collective settings of Ghana, prospective research participants may request the researcher to first seek approval from the chief/elders of the community, or from the head of the household, or in the case of a married female participant, from the husband.	In most rural communities of Ghana, there are clearly outlined procedures to follow to obtain permission from the gatekeepers of the community (i.e., chief and elders) before participants can be approached in their homes to be recruited.	The author urged prospective researchers to carefully explore and respect the cultural values and practices of community members and observe locally-defined ethical values and principles when conducting community-based participatory research in rural African settings to minimise ethics dumping and safeguard the integrity of their research.
8	Araali, B. B (2011). Perceptions of research assistants on how their research participants view informed consent and its documentation in Africa	Discrepancies between African socio-cultural values and research ethical practices derived from Western values – collectivistic versus individualistic viewpoint of personhood and autonomy.	Requesting participants’ signatory on informed consent documentations could engender fear that they are committing to the unknown, deceit, or treachery.	Researchers and research institutions should incorporate African values into the informed consent and research processes when conducting research in the highly communal Africa contexts, and collaborate to generate clear guidelines that satisfy both the interests of ordinary Africans (particularly the rural and uneducated population) and standard research ethical principles.
9	Behrens (2018). A critique of the principle of ‘respect for autonomy’, grounded in African thought	‘Respect for autonomy’ is concerned only with individual decision-making, with the purported right of individuals to make choices about their health and life entirely on their own. However, in most of Africa, the notion of autonomy is problematic because community is prized and individuals are mostly bound up with their communities.	For many Africans, the principle of ‘respect for autonomy’ is problematic because it fails to recognize the important fact that individuals are embedded within communities.	The author argues that ‘respect for persons’ is a more appropriate principle, as it is able to acknowledge both individual decision-making and the essential relationality of persons. The author proposes a relational definition of personhood that distinguishes between persons with agency and persons without agency, arguing that we have different moral obligations to these distinct categories of persons
10	Brear, M (2018). Ethical research practice or undue influence? Symbolic power in community-and individual-level informed consent processes in community-based participatory research in Swaziland.	Gatekeepers/leaders wield some “symbolic power”, and their involvement in the informed consent process could coerce participation. However, this is minimized when informed consent is introduced and explained to necessarily (and strictly) require individual autonomy and rights.	Participants ascribed different (often contrary) views and meanings to ‘voluntariness’ of a research study. The concept of voluntary informed consent was novel and culturally inconsistent with norms in the study settings.	In African contexts where individual rights and autonomy are foreign concepts, and when community-level consent is required, the informed consent process must continually enquire from participants that they agree to continue participating.
11	Chukwuneke et al. (2014). Global bioethics and culture in a pluralistic world: How does culture influence bioethics in Africa?	In most Africa, people draw on the views and inputs of others in their decision-making, which starkly contrasts the individual decisional authority emphasized in informed consent and research.	None discussed	Researchers and bioethicists should be contextual, pluralistic, and respectful of the cultural diversity of non-Western cultures in their research engagements.
12	Diallo et al. (2005). Community permission for medical research in developing countries.	The gatekeeping and functional roles of community leaders require researchers to visit leaders in their homes to solicit their support and inputs before study commences.	None discussed	For health-related research, researchers should develop formal agreement with traditional health care providers for collaboration on the research project.
13	Dunin De Skrzynno and Di Maggio (2018). Surgical consent in sub-Saharan Africa: a modern challenge for the humanitarian surgeon.	The majority (72%) of female patients who accessed emergency surgical procedures requested the presence (and agreement to treatment) of another member of the family, usually the husband; on the other hand, only 5.5% of male patients requested the input of another person.	Informed consent is influenced by cultural background, family structure, socioeconomic status, religion and education. Stakeholders’ views are important to patients and for the treatment process.	Consenting practice must be integrated in a culturally appropriate manner into each individual society, particularly in contexts where communal thinking and living is the norm.
14	Frimpong-Mansoh, A (2008). Culture and voluntary informed consent in African health care systems	The individual-based informed consent model compromises customary practices in some African communities that require researchers to consult with, and obtain permission from, community leaders (such as village councils, chiefs, queen mothers, clans, and family elders) before approaching potential research participants.	None discussed	Most African communities require researchers to obtain approval from leaders and elders of the community, in addition to seeking informed consent at the individual level – which is an inherent multi-step gatekeeping strategy to protect members from undue exploitation and safeguard their well-being from potential invasive and harmful experiments.
15	Gebresilase et al. (2017). Rapid ethical appraisal: A tool to design a contextualized consent process for a genetic study of podoconiosis in Ethiopia.	None discussed	Participants suggested that researchers should approach prospective participants in the presence of known and trusted community members, including religious and *kebele* leaders, health extension workers, patient association leaders, local NGO staff, the ‘1-to-5’ networking scheme (a government-created structure where one member networks with five other people and leads the group to discuss societal issues), and *Idir* (locally established traditional self-help financial associations).	Participants (i.e., researcher/IRB members) argued the need for a pre-test of the consent form and information sheet in the study site to assess its suitability and appropriateness, prior to the main study. Participants also urged researchers to explore and understand the concerns of, and incorporate the values of, the local people into the informed consent and research processes in order to satisfy the interests of the researcher and the participants.
16	Gondwe et al. (2022). Guardians and research staff experiences and views about the consent process in hospital-based paediatric research studies in urban Malawi: A qualitative study.	Guardians in the studies felt that their family members were neglected in the consenting process.	Research staff reported that they had problems obtaining consent from guardians when their spouses or family members were not present.	There is need to adopt a flexible, continuous consent process, and utilize an individual approach to information provision prior to consenting, rather than a group approach.
17	Jegede, S (2009). African ethics, health care research and community and individual participation.	The African communal sociocultural structure opposes individual autonomy underpinning standard informed consent, making it difficult to apply an autonomy-based model for research in most of Africa.	It is problematic to apply the Western concept of autonomy to conduct research with human subjects in Africa, giving the disparity in the worldviews of personhood shared by the two divides.	Community participation should be promoted for health research in Africa, although prospective participants should make the final decision about whether or not to participate in any research
18	Jeko et al. (2012). Obtaining informed consent in non-Western contexts: Reflections on fieldwork experiences in Zimbabwe.	While the standard informed consent protocol emphasizes freedom of choice and personal decision-making, however in the more communal settings of Zimbabwe (and Africa), decisional authority on important matters tends to lie outside the remit of the individual, and instead spread across various centers of power in social formations.	Requesting for signatory on the consent form could be misconstrued by participants for a lack of trust, which could potentially constrain the relationship between the research team and participants.	It may be difficult to obtain first-person informed consent in parts of Africa, owing to the residual communalistic orientation of the communities. Researchers should be more culturally sensitive by seeking to reconcile the ethos of particular communities and the ethical principles for informed consent.
19	Kengne-Ouafo et al. (2014). Perceptions of consent, permission structures and approaches to the community: A rapid ethical assessment performed in North West Cameroon.	The current informed consent process neglects the invaluable role of the *fon* (local traditional authority) in the community entry and informed consent processes; most participants favored the argument that consent for biomedical research should first be given by the *fon.*	Even with appropriate ethical approval and permission from political and relevant stakeholders, community members expect researchers to first contact the *fon* to fulfill the community entry process. This engagement offers the *fon* and the council of elders the opportunity to appraise the objectives, potential harm, and benefits of the study to community members – which is their core function as gatekeepers.	Participants acknowledged the permission-giving structures in their communities, and argued that the rapid ethical assessment approach would guide the conduct of context-appropriate, ethically-sound informed consent processes for future studies in North West Cameroon.
20	Marshall et al. (2014). Voluntary participation and comprehension of informed consent in a genetic epidemiological study of breast cancer in Nigeria.	A considerable number of female participants reported either informing, or asking permission from, their husbands to enroll in the study, although they emphasized their ability to make decisions for themselves – whether or not they inform their husbands about their intentions.	Participants argued for different approaches, with more allowance for gatekeeper involvement in rural settings.	Further research needed to explore complex issues associated with the informed consent process, including communication of information, comprehension, decisional authority and voluntary participation.
21	Molyneux et al. (2005). ‘Even if they ask you to stand by a tree all day, you will have to do it (laughter)…!’: community voices on the notion and practice of informed consent for biomedical research in developing countries.	Standard informed consent procedures exclude community leaders, who are gatekeepers and custodians of the communities.	With community-based biomedical research, participants, such as mothers with very sick children, may not be in the right frame of mind, or would not wish to understand or be bewildered by potentially invasive biomedical procedures.	There is a growing need for context-appropriate informed consent procedures to facilitate the research process at the community level that reflect the ideals and values of the communities involved in the study. Participants suggested that informed consent should be autonomous at the individual level, in addition to the community level provided by gatekeepers.
22	Moodley, K (2002). HIV vaccine trial participation in South Africa–an ethical assessment.	The individual-focused consent framework constrains the rights of prospective research participants in many African countries, potentially rendering the research process unethical.	There is a compelling need for researchers to secure family consent in addition to individual consent in biomedical research.	Ethical principles and research guidelines must be adapted and applied to the community in which the research will be done, taking account of their traditions and honoring their practices.
23	Moyer et al. (2022). Social complexities of informed consent and assent among young males undergoing voluntary medical male circumcision in Eswatini.	Often, important decisions, including health-related decisions, are made by groups at the extended family level, but also on other bases such as neighbourhood or religious congregation.	Practically, respect for young males’ rights and decision-making in consenting to (health-related) research is limited by complex social, economic and political realities	The research informed consent models need to examine the role and influence of social, economic, and political factors on research and to incorporate them into existing informed consent frameworks.
24	Nyirenda et al. (2020). Structural coercion in the context of community engagement in global health research conducted in a low resource setting in Africa.	Some village leaders coerced participation with normalized threats and thus compromise ethical research practice, although their involvement could also boost members’ acceptance and participation in emergency health research and interventions.	Some village leaders enacted and enforced certain by-laws by using threats to foster research participation	Researchers need to balance efforts to improve informed participation through community engagement, with the need to ensure voluntary participation in research
25	Osamor, P. E and Kass, N (2012). Decision-making and motivation to participate in biomedical research in southwest Nigeria.	Informed consent in this community is understood and practised as a relational activity that involves others in the decision-making process, rather than as an individual engagement.	None discussed	Researchers in West Africa should seek community consent before approaching individuals for their consent to participate. Spousal permission may not necessarily diminish voluntary participation in research in West Africa.
26	Princewill et al. (2017). Factors affecting women's autonomous decision making in research participation amongst Yoruba women of Western Nigeria.	Obtaining a first-person informed consent for women's participation in research is somewhat problematic in most African settings, because culture promotes the idea that men are ‘keepers’ for other family members.	None discussed	African researchers and bioethicists should generate and evaluate culturally appropriate and workable recruitment methods for research participation. These new methods should encourage the autonomous decisions of women who wish to participate in research.
27	Ramabu, N.M (2020). Whose autonomy is it? Botswana socio-ethical approach to the consenting process.	Consenting process differed according to age, role, and location. Policy makers, practitioners, and participants from the city opted for individual consent process; whereas participants from the village requested researchers to obtain permission from community leaders prior to their participation.	None discussed	Respect for persons in Botswana is viewed and practised relationally, suggesting that current bioethical frameworks need to take into account the socio-ethical respect for persons in order to conduct ethical research in the Botswana context.
28	Rashad et al. (2004). Obtaining informed consent in an Egyptian research study.	The notions of autonomy and individual-based informed consent somewhat contrast Egyptian collectivistic cultural values.	In most Egyptian settings, decision-making is commonly deferred to male figures, including father, husband, or teachers – who must be consulted as part of the consent process.	Researchers should be sensitive to cultural understanding and religious beliefs of the local people and tailor the consent process to embrace these dynamics.
29	Shaibu, S (2007) Ethical and cultural considerations in informed consent in Botswana	Some individuals prefer to have other family members, who they consider to be a part of their lives, also sit in the recruitment, informed consent, and interview processes.	None discussed	Rather than forcefully apply the Western, individual-focused framework, scholars in non-Western countries should carefully consider a diversified framework that acknowledges and blends the cultural values and practices of the African peoples with the science and principles underpinning the standard research ethical codes, as appropriate
30	Tekola et al. (2009). Tailoring consent to context: Designing an appropriate consent process for a biomedical study in a low-income setting.	Exclusion of locally trusted individuals/gatekeepers, who are elected to safeguard the interest of the community, from the informed consent process. Individual consent should also be preceded with community sensitization to optimize communication, comprehension, and acceptability of informed consent.	Most participants (especially those unable to read and write and unaccustomed to signing documents) did not favor the use of written information sheets and consent forms, and instead, showed preference for oral consent.	Researchers should tailor and evaluate the effectiveness of consent processes to facilitate the provision of appropriate information in a comprehensible manner and in supporting voluntary decision-making on a study-by-study basis.
31	Tindana et al. (2006) The informed consent process in a rural African setting: A case study of the Kassena-Nankana district of northern Ghana	The universal informed consent framework neglects the essential role of community leaders in the consent and recruitment processes.	Some participants preferred a verbal consent process, primarily because they could not read the consent forms which were written in the English language	When working in the more communal settings of Ghana, the research team should firstly engage with the traditional authorities (i.e., chief and his council of elders) for permission before approaching households to invite prospective participants.
32	Visagie et al. (2019) Informed consent in Africa – Integrating individual and collective autonomy	The principlist biomedical-derived framework that underpins the informed consent is not well-suited for research involving rural African communities, as the rigid guidelines are not appreciative of the sociocultural dynamics in these communities.	The informed consent process significantly misaligns with the decisional making structure in rural collectivistic African settings, who are more relationally interconnectedness rather than individualistic.	The authors argued for, and proposed, an integrated informed consent approach founded on Afro-communitarianism for use by social science researchers in Africa. Consent should be an ongoing process that builds people's capacity to consent, in recognition of their cultural authority structure, customs, norms, without disregarding personal capacity.
33	Vreeman et al. (2012a). Community perspectives on research consent involving vulnerable children in Western Kenya.	Participants advocated for the involvement of caregivers (in the case of research involving minors), community leaders, and even community assemblies to actively participate as stakeholders in the consent process.	None discussed	Researchers should engage with communities in an informative consent process that includes careful discussion of procedures, risks, and benefits and modify the research process (including the informed consent) to meet the understanding and needs of the target group.
34	Vreeman et al. (2012b). A qualitative study using traditional community assemblies to investigate community perspectives on informed consent and research participation in western Kenya.	Researchers needed to firstly collaborate with the communities via *baraza* – a culturally accepted public assembly for the purpose of community dialogue in order to explore community members’ understanding of research, informed consent, and community consent and to generate context-appropriate informed consent and research approaches.	None discussed	Traditional community forums, such as *mabaraza* in East Africa, can serve as a medium to further engage communities in community consent and other aspects of research. Participants advocated for a community consent approach to complement individual informed consent, rather than as its replacement.
35	Zulu et al. (2019). The challenge of community engagement and informed consent in rural Zambia: An example from a pilot study.	An array of structural factors and cultural norms about the value of marriage, local notions of social status, and perceived trustworthiness or credibility of the researchers weigh in on the consent process.	Participant recruitment and the consent processes are significantly influenced by structural and contextual factors such as poverty and low literacy rates, which constrain their understanding and participation in research activities.	Researchers must carefully consider the complexity of local values and structures that may impact people's capability to consent or not consent to a study in an informed manner.

#### Subversion of Gatekeepers’ Functions

Seven studies reported that the standard IC model, with its emphasis on individualism, essentially neglects the important functions of community leaders and gatekeepers in research, particularly in the rural, socioeconomically disadvantaged settings of Africa (Diallo et al., 2005; Gondwe et al., 2022; Kengne-Ouafo et al., 2014; Molyneux et al., 2005; Tekola et al., 2009; Tindana et al., 2006; Vreeman et al., 2012a). Studies find that gatekeepers, including chiefs and council of elders, Assembly representatives, Unit Committee members (e.g., for health, education, sanitation, etc) are elected, often politically, to govern and preserve the culture of the group, to spearhead developmental activities and well-being of residents, and to protect members from exploitation (Appiah, 2021). However, the involvement of gatekeepers, such as the head of a household or husband of a prospective married female participant in the decision-making process could be construed as a potential source of interference and considered a violation of the principle of respect for autonomy that underpins the IC model (Azétsop & Rennie, 2010; Ikuenobe, 2018). By backing their claims with evidence from anthropological research that suggests that much of African communities are collectivist, which are governed by elected leaders to protect the interest of members (Chukwuneke et al., 2014; Gyekye, 2014), the studies contend that a strict adherence to the principle of autonomy inferentially alienates the important (protective) functions of gatekeepers, such as from the exploitation of crude researchers and to minimize ethics dumping. Of note, the majority of studies arguing the subversion of gatekeepers’ roles did not recommend a blanket, community level consent as sufficient approach to consenting, but instead, suggested a diversified, multi-step approach that seeks consent at both community and individual levels. For instance, a study in Kenya that examined community members’ perspectives on the IC procedures, and their suggestions on how to conduct context-appropriate IC process, found a widespread consensus by community members that chiefs and elders should give approval to researchers to conduct the study in the community, but that households and individuals should provide autonomous IC (Molyneux et al., 2005). In a study in Ethiopia that explored the perspectives of community members on the procedure to follow to gain consent, participants recommended that researchers approach prospective participants through, or in the presence of, locally trusted individuals (Tekola et al., 2009). This notion was also echoed by participants in Burundi (Dunin De Skrzynno & Di Maggio, 2018), Cameroon (Kengne-Ouafo et al., 2014), Ghana (Tindana et al., 2006), Kenya (Vreeman et al., 2012a), Malawi (Gondwe et al., 2022), and Mali (Diallo et al., 2005).

Despite the reported claim of the functional roles of community leaders and gatekeepers in research in some African contexts, we retrieved three studies that critically questioned the implications of gatekeepers’ involvement in research, implicating them, instead, of abuses of power and undue coercion of community members to participate in research. For instance, a qualitative ethnographic study that explored the ethical issues arising from community engagement in Malawi found that although the involvement of community leaders in research is considered culturally appropriate (that could possibly boost participation) in most Malawian settings, authoritarian leadership structures could potentially coerce and undermine individuals’ autonomy to make voluntary decisions about participation (Nyirenda et al., 2020). In Eswatini, research was conducted to interrogate the social relations of IC in community-based participatory research in a rural Swazi community, and to investigate how obtaining community-level consent influenced community members’ autonomy to provide individual-level consent (Brear, 2018). The study found that whereas participants recognized the essence of involving community leaders in the research process, they also acknowledged that the “symbolic power” of community leaders has the potential to constrain individual agency. Similarly, among a sample of Yoruba women in Nigeria, Princewill and colleagues found that a combination of patriarchal, religious, and cultural factors colludes to adversely impact on the autonomy of women with respect to research participation (Princewill et al., 2017). The study further revealed that although men feel they were protecting women by making decisions for them, the women interpreted this ‘protection’ as a stratagem by the men to limit their autonomy, suggesting that authoritarian community leaders and male figures in highly patriarchal societies may despise the requirements of the individual-based, autonomy-driven IC model and argue its inappropriateness in their context.

In two studies, participants showed a mixed preference for either individual or community level consenting for research participation. In exploring the ethical issues and challenges associated with approaching communities and gaining IC in Cameroon, participants generally showed preference for a centralized permission-giving structure that they expect researchers to follow (Kengne-Ouafo et al., 2014). However, a considerable number of participants with higher levels of educational attainment objected to this structure, and instead, opted for an individual-based consenting model. Similarly, in Botswana, participants (comprising policy makers and practitioners) in the village and urban areas both showed preference for an individual autonomy model of consenting when asked to suggest the IC process for bioethics research in Botswana communities. On the other hand, whereas caregiver participants in the cities exercised individual autonomy to participate in the study, their counterparts in the villages requested that permission be obtained from community leaders before they could participate (Ramabu, 2020). Findings suggest a co-existence of collectivist and individualist orientations in parts of Africa, mostly with characteristics of individualism among educated individuals dwelling in the urban settings, and collectivism among people in rural settings.

### Recommendations to Manage IC-Related Challenges

#### Adopt a Multi-Step Approach to IC

Several studies (18) in this review recommended the adoption of a multi-step approach, that solicit consent at both the community and individual levels (Abdel-Messih et al., 2008; Behrens, 2018; Brear, 2018; Dunin De Skrzynno & Di Maggio, 2018; Frimpong-Mansoh, 2008; Jegede, 2009; Marshall et al., 2014; Molyneux et al., 2005; Moodley, 2002; Osamor & Kass, 2012; Princewill et al., 2017; Ramabu, 2020; Shaibu, 2007; Tekola et al., 2009; Tindana et al., 2006; Visagie et al., 2019; Vreeman et al., 2012a, 2012b; Table 2). Rather than operationalize the IC to be solely individual-centered, the studies recommended a diversified approach, wherein IC is sought both at the community and household levels, if required, in addition to individual level consent. The studies argued that this pluriversal approach could essentially satisfy the cultural norms and expectations of the communities, as well as the autonomic requirement of the IC model. When asked about their perspectives on the IC procedures and suggestions for context-appropriate IC process in Kenya, participants recommended that researchers should allow gatekeepers to provide IC at the community level, while individual participants provide independent, autonomous consent (Molyneux et al., 2005), thus operationalizing the IC as a multi-step process rather than a once-off exercise. Frimpong-Mansoh (2008) contends that most African communities require researchers to obtain approval from leaders and elders of the community, in addition to seeking IC at the individual level. Inherently, a multi-step approach to IC would also serve as a multi-step gatekeeping strategy to protect members from undue exploitation and safeguard them from potential invasive and harmful experiments. Rather than forcefully apply the individual-focused IC model, Shaibu (2007) argued that researchers in collectivist social settings should carefully consider a diversified IC model that acknowledges and blends the cultural values and practices of the African people with the science and principles underpinning the standard research ethical codes, as appropriate. That a blended, multi-step approach to IC is more appropriate for much of African communities has also been vociferously advocated in the literature by scholars in Botswana (Ramabu, 2020), Burundi (Dunin De Skrzynno & Di Maggio, 2018), Ghana (Appiah, 2021; Tindana et al., 2006), Eswatini (Moyer et al., 2022), Ethiopia (Gebresilase et al., 2017), Kenya (Vreeman et al., 2012b), Malawi (Gondwe et al., 2022; Nyirenda et al., 2020), Nigeria (Osamor & Kass, 2012; Princewill et al., 2017), and South Africa (Akpa-Inyang & Chima, 2021; Moodley, 2002).

#### Conduct a Rapid Ethical Assessment

We found six studies that either recommended or reported on the design, implementation, or evaluation of a *rapid ethical assessment* to examine plausible ethical issues and challenges akin to a particular population group, prior to conducting a study (Abay et al., 2016; Addissie et al., 2014, 2016; Gebresilase et al., 2017; Kengne-Ouafo et al., 2014; Zulu et al., 2019). The rapid ethical assessment involves the conduct of a brief qualitative research to familiarize with the context, explore local contextual factors that could potentially constrain the research process, and to identify ways to approach communities and manage potential ethico-cultural issues related to the IC and research process. By conducting a rapid ethical assessment ahead of their study, researchers in Ethiopia found that only a fraction of study participants was satisfied with the current standard consent process (with its emphasis on individual autonomy), suggesting gaps with the consent process that need to be further explored and resolved (Addissie et al., 2014). In north west Cameroon, participants acknowledged the permission-giving structures in their communities, and intimated that the rapid ethical assessment approach would guide the conduct of context-appropriate, ethically-sound IC processes for future studies (Kengne-Ouafo et al., 2014). Again, in Ethiopia, participants (i.e., researchers and IRB members) emphasized the need for a pre-test of the consent form and information sheet in the study site to assess its suitability and appropriateness, prior to the main study (Gebresilase et al., 2017). In the same study, participants implored researchers to explore and understand the concerns of the local population and to incorporate their values and beliefs into the IC and research processes in order to satisfy the interests of both stakeholders. Abay and colleagues argued that, overall, rapid ethical assessment could potentially reveal important sociocultural issues which could serve as invaluable resource in modifying the consent information documents to suit the study context (Abay et al., 2016).

#### Generate an African-Centered IC Model

Sixteen studies noted that because the standard IC model is essentially based on the Western concept of libertarian rights-based autonomy, its theoretical underpinnings do not fit well with the African notion of *being* or *personhood*, which emphasizes more on an individual's relationality with others (Abdel-Messih et al., 2008; Akpa-Inyang & Chima, 2021; Appiah, 2020, 2021; Araali, 2011; Chukwuneke et al., 2014; Diallo et al., 2005; Gebresilase et al., 2017; Jeko et al., 2012; Marshall et al., 2014; Molyneux et al., 2005; Moyer et al., 2022; Tekola et al., 2009; Visagie et al., 2019; Vreeman et al., 2012a; Zulu et al., 2019). These studies argued the need for a pluriversal IC model that takes into account the values, beliefs, and practices of the African peoples to facilitate the conduct of research with human subjects in Africa. Molyneux et al. (2005) noted the growing need for context-appropriate IC procedures to facilitate the research process at the community level that reflect the ideals and values of the communities involved in the study. Likewise, Abdel-Messih et al. (2008) implored researchers and research administrators to collaborate with local communities to develop culturally-competent and diversified recruitment and consent procedures that acknowledge and incorporate the cultural, political, and social practices of the local context. Tekola et al. (2009) also entreated researchers in Ethiopia to tailor and evaluate the effectiveness of the consent process in order to optimize comprehension and participants’ decision-making on a study-by-study basis. In Ghana, Appiah (2021) argued for a more pluriversal approach to IC and urged researchers, universities, research institutions, and institutional review boards in Africa to collaborate with local communities and stakeholders to design and implement context-appropriate IC models and procedures to build mutual trust and respect, increase interest and participation, prevent ethics dumping, and safeguard the integrity of the research process.

## Discussion

This review maps and critically evaluates studies that report on the ethico-cultural and implementation challenges associated with the standard, individual-autonomy driven IC model in the African context. The review also examined suggestions and recommendations in the literature to manage these challenges.

At the theoretical level, ethical principles, values, and codes are largely conceptualized to reflect the sociocultural values and belief systems of the context in which the codes have been developed (Killawi et al., 2014; Leong & Lyons, 2010). This suggests that some values underpinning (research) ethical guidelines may not necessarily have universal utility. Ruiz-Casares argues that applying the Western standards for voluntary individual decisions in non-Western, more collective community settings, which mostly emphasize the needs of groups over individuals, can be problematic (Ruiz-Casares, 2014). This review found that although efforts to protect the autonomy of prospective participants in the African context are important and laudable, a stringent prioritization of individual autonomy over and above other moral values such as collective autonomy or decision-making, are inappropriately narrow. Although some researchers in Africa acknowledge the disparities between the requirements of the traditional IC model and the cultural values and practices of the local population, they refrain from making revisions to the consent forms, partly because of the bureaucracy involved in making these amendments to the research protocol (Addissie et al., 2016).

A cardinal requirement of the IC is that cognitively capable adults be allowed to decide on whether or not to participate in research or clinical treatment, thus upholding their right to self-determination (Kadam, 2017; Nijhawan et al., 2013). Yet, the findings of this review suggest that in most African communities, members of the family (and leaders of the community) primarily function as gatekeepers and support systems – who also have politico-culturo-moral obligations to protect the interests of community members, and support them to weigh their decisions and choices for their personal and collective good. Overall, the findings suggest that the decision-making process in parts of Africa is largely multi-layered rather than a solely personal endeavor. Views and insights solicited from, and shared by, close others are principally intended to provide additional perspective to help the member to further appraise their decisions and choices, and not necessarily to impede or interfere with the decision-making process (Visagie et al., 2019).

A substantial number of the reviewed studies suggested that the standard IC model essentially neglects the important gatekeeping functions of community leaders and other political authorities in the communities. In most rural, collectivist settings of Africa, the chief and other gatekeepers are culturally mandated to welcome researchers into the community, evaluate the potential risks and benefits of the study, and when satisfied, to inform community members about the presence of the researchers in the community and to request members’ support for the research activities (Appiah, 2020, 2021; Msoroka & Amundsen, 2018; Tindana et al., 2006). The role of gatekeepers, particularly in the rural settings, serve to protect the interest of community members, some of whom may have low educational attainment, are socioeconomically disadvantaged, and potentially vulnerable. There was, however, evidence of potential abuse of power and coercion of members to participate in research activities by authoritarian leaders in highly patriarchal societies (Brear, 2018).

We note the argument advanced by some researchers regarding the unethicality of community consent and the involvement of gatekeepers in the IC process. For instance, Andoh (2016), by taking a more developmental perspective, dismissed the possibility of cultural influences on the IC process and surmised that other socioeconomic factors, such as low literacy and poverty, are responsible for the challenges associated with implementing the IC in some African contexts. Andoh opined that community consent should be subverted for community dialogue and community education in research in Africa, and further argued for mechanisms to improve comprehension and capacity of “vulnerable populations living in extreme poverty and affected by high levels of ignorance” (Andoh, 2016, p. 122). We note, however, that in a later work that critiques the universality of biomedical ethics, Andoh argues that ‘genuine development of bioethics in Africa must be rooted on core communitarian ethical principles and must rest upon the innate authentic African communitarian theories’ (Andoh, 2016). Unquestionably, communal consent and the involvement of gatekeepers in the IC process poses a fundamental challenge to the individualistic oriented IC model (Mkhize, 2006; Segalo & Molobela, 2019). Yet, the studies in this review contend that if ethical guidelines are primarily enacted to conduct ethical research, then the principle of respect for persons should also be extended to demonstrate respect for the cultural and customary beliefs, values, and expressions of the people in the communities that are researched (Behrens, 2018; Visagie et al., 2019). Overall, the evidence suggests that gatekeepers and community leaders could contribute to research activities by facilitating the community entry process, by helping the research team to navigate the communities, and by assisting researchers to identify eligible or vulnerable individuals and groups for research. Nonetheless, some authoritarian leaders might employ their power to compel individuals to participate or to hinder them from doing so, particularly in highly patriarchal societies (Brear, 2018).

Much of the reviewed literature proffered diverse strategies to guide researchers to overcome specific ethico-cultural and implementation challenges. A multi-step approach to IC that allows researchers to seek consent at both the community-, household-, and individual-levels was widely recommended as ideal for research with human subjects in the relatively collectivistic African context. The suggested multi-step approach is argued to have the potential to ensure that the researcher meets communal norms and requirements for community entry, while also granting individuals the space to express their cultural beliefs and values and to involve others in the decision-making process, if they so desire. This approach enables the researcher to establish that the final decision rests with the prospective participant (Ikechukwu Osuji, 2014), thus satisfying both the basic requirements of IC and the cultural milieu of the local population. Similarly, in their effort to create awareness and guide researchers to envision and manage research implementation challenges in more appropriate ways, researchers have advocated for, designed, evaluated, and propagated an ethnographic assessment, termed as rapid ethical assessment (Abay et al., 2016; Addissie et al., 2014, 2016; Gebresilase et al., 2017; Kengne-Ouafo et al., 2014). This pre-study qualitative assessment is intended to guide researchers to discover, describe, and respond to potential ethico-cultural issues (e.g., beliefs and cultural characteristics and practices) akin to a population group. While not the ultimate panacea to the challenges posed by the ideological and ontological dichotomies between the individualistic-oriented IC and the collectivistic African worldviews, the rapid ethical assessment can potentially minimize ethics dumping and critically ensures that the design of IC procedures considerably aligns with the traditional cultural values and practices of the target population.

The literature is replete with suggestions for African-based researchers to collaborate in generating an African-centered IC model to facilitate research with human subjects in Africa. This call is largely premised on the argument that if the principle of respect for persons or autonomy appeals to the researcher to respect the individuality and rights of research participants, then it is even more important that this right is extended to respect the cultural values and beliefs of the local population who agree to participate in research (Behrens, 2018; Visagie et al., 2019). Visagie and colleagues took a more pragmatic view, arguing that the principles underpinning the standard IC framework is particularly suited for biomedical research and practice (Visagie et al., 2019), and that there is urgent need for a framework that is tailored towards the goal and scope of social science research in Africa, where collective (e.g., family- and group-based) decision-making is ubiquitous.

### Implications and Recommendations for Research and Practice

The findings of this review have several important implications for research and clinical practice in some African settings. Firstly, many African philosophical perspectives and ontologies conceptualize the individual as a distinct being who also maintains strong ties and shares their identity and self with the community (Gyekye, 2011). The main challenge with the individual-based IC model in some African communities arises from the fact that the IC framework is heavily aligned with the Western-individualistic worldview of personhood, which starkly contrasts with the collectivistic, inter-relational view of personhood practiced in most parts of Africa (Gyekye, 2011; Mpofu, 1994). The evidence from this review suggests that people in collectivist societies regularly confer with significant others and members of the community as a secondary mechanism to solicit views and affirm their decision, if they were unsure. Furthermore, gatekeepers (e.g., chiefs, community elders, unit committee members) also play a crucial role in protecting community members, many of whom have little or no formal educational attainments, from deceit, exploitation, and ethics dumping (Appiah, 2020, 2021). Again, some socioeconomic factors, such as low literacy, which are often overlooked in the discourse, could constrain a person's capacity to make an informed decision about a specific subject matter that is unfamiliar to them, even though they are capable of making decisions about issues of daily living.

In line with the findings of this review and in consensus with the extant literature (Blanch et al., 2008; Nembaware et al., 2019; Tiffin, 2018), we strongly endorse the adoption of a multi-step approach to IC in highly collectivistic African communities. Our support for this approach aligns with a broader scholarly consensus advocating for nuanced and culturally sensitive practices to facilitate research with human subjects in the relatively collectivistic context of Africa. Furthermore, the findings of the present review acknowledge the distinctions in preferences for a multi-layered IC approach within certain African contexts. These variations, often influenced by socio-cultural factors and educational backgrounds (Appiah, 2021; Heerman et al., 2015; Kiguba et al., 2012), underscore the imperative of a flexible, multi-step IC model. The diverse perspectives, in themselves, are critical for ensuring inclusivity and cultural sensitivity in the IC process, further necessitating the need for an adaptable, context-tailored IC model for the African peoples.

Secondly, an essential criterion for autonomous decision making, besides the person being fully aware of making a decision in the face of adequate information to inform the decision-making, is that the decision-making process must be free from controlling influences, which is operationalized to encompass all premeditated actions that contravene the beliefs, values, and desires of the decision-maker that have the potential to sway the decision (Nelson et al., 2011; Papillon & Rodon, 2017). Controlling influences typically include coercion, manipulation, lying, threats, nondisclosure of information, or suggestions of urgency in non-urgent situations (Beauchamp, 2010; Nelson et al., 2011), yet, it may be difficult, if not inappropriately narrow, to categorically equate a person's expression of their cultural norm or their quest to seek the view of another to inform their decision on whether or not to participate in research, to a premeditated controlling stratagem. We agree with Visagie et al. (2019) that the concept of individual autonomy – which drives the IC framework – significantly misaligns with the decision-making structure in much of Africa, which is relatively collectivistically socially oriented. This review finds that the autonomy-driven IC potentially creates intercultural power inequalities and colonial legacies that need to be explored and addressed to facilitate an ethical research process that also acknowledges the cultural practices, values, expectations, and contributions of the local population group, while also safeguarding the integrity of the research process.

Thirdly, although models and frameworks that provide research guidelines from global perspectives are important, they are inappropriately narrow in envisioning, managing, and providing guidance for specific contexts, particularly in LMICs, where the research process presents a distinct set of complexities (de Vries et al., 2015). Typically, much attention is spent ensuring that the research methodology and procedures adhere to the standard, universal ethical guidelines approved by the ethics committees. However, to the extent that an individual's decisional trajectory and their conceptualization, understanding, and behavioral expressions of phenomena are partly determined by their sociocultural orientations (Han & Ma, 2015; Matsumoto, 2007), the influence of cultural norms and practices on the research process cannot be ignored. Findings from the present review underscore the need for researchers to carefully explore and integrate context-specific factors and cultural values and norms of the local population into their IC and research procedures.

Fourthly, the reviewed studies revealed some nominal aggregations in the findings across countries, with the main themes cross-cutting all blocs of the continent. For instance, in Kenya, east Africa, there was a widespread agreement by community members that chiefs and elders should give approval to researchers to conduct the study in the communities, but that households and individuals should provide autonomous IC (Molyneux et al., 2005). However, in Cameroon, a central African country, despite objections by some educated individuals, the majority of participants described a centralized permission-giving structure in their communities that they expect researchers to abide by, including through the health center and the *Fon* (traditional leader) (Kengne-Ouafo et al., 2014). Yet, in Eswatini, a southern African country, participants recognized the essence of involving community leaders in the IC process, but also noted that gatekeepers could coerce participation (Brear, 2018). On the other hand, in Mali, west Africa, participants recommended a community level consent, in addition to individual level consent, noting that community level consent could facilitate the process of disclosure for individual IC (Diallo et al., 2005). Taken together, these findings suggest marked similarities and peculiarities in the requirements for ethical research across Africa. Therefore, it is crucial to approach the implementation of IC frameworks in African research with careful consideration of the diverse values inherent to African peoples. The findings, therefore, emphasize the importance of considering the unique cultural and contextual factors within each African community, promoting ethical research practices that align with the values of the populations under study. Overall, the present review's findings implore researchers to recognize the need for nuanced, localized approaches to IC practices across African communities.

Lastly, the concept of an African methodological approach to research also encompasses the realm of bioethics. For instance, Barugahare (2018) argues that while advocating for an African approach to bioethics is commendable, it is accompanied by certain methodological challenges that warrant deeper exploration and consideration. The methodological insights presented by Barugahare (2018) to address these challenges are in concordance with the findings and recommendations articulated within this review. Firstly, researchers are encouraged to recognize the significance of African cultural identity when examining the broader ethical implications, thus ensuring inclusivity and objectivity in bioethical discussions. This approach aligns with the recommendations outlined in this review, advocating for researchers to adopt a balanced stance on consent that both respects cultural values and addresses ethical concerns. Secondly, Barugahare argues that evaluating bioethical principles based on their inherent merit fosters a culturally relevant yet morally sound approach to healthcare challenges. This underscores the necessity of adopting a multi-step approach to IC in Africa, wherein principles are impartially evaluated to accommodate cultural nuances and uphold ethical integrity. Thirdly, instead of outright rejection of Western bioethics, Barugahare (2018) suggests complementing existing frameworks with African moral insights, facilitating the adaptation of global standards while honoring cultural norms. These insights are congruent with the findings of the present review, which advocate for a rapid ethical assessment process to tackle the implementation challenges of individual-based IC frameworks in African contexts. Finally, Barugahare (2018) argues that by prioritizing contemporary realities in bioethical discussions, we can develop responsive frameworks to address evolving healthcare challenges. This highlights the imperative of developing an African-centered consent paradigm that integrates contemporary healthcare methodologies and emerging technological advancements. Such an approach resonates with the overarching focus on addressing pragmatic and implementation obstacles within IC frameworks.

## Conclusions

This review is the first to provide an overview of the ethico-cultural and implementation challenges associated with the individual-based, autonomy-driven IC model in the African context, with suggested strategies to manage the challenges. Overall, the study finds that the individual-based IC presents considerable ethical, cultural, and practical challenges when administered for research in the relatively collectivistic settings of Africa. This finding does not in any way suggest that researchers in Africa should waive or disregard the traditional research requirement for individual decision-making with respect to the IC. Instead, the evidence contributes to the ongoing discussions and calls for a re-examination of the appropriateness of the autonomy-oriented IC model in the relatively collectivistic social context of Africa. A multi-step approach, where consent is sought at both the community and individual levels, was widely recommended. This model, among others, is envisaged to help prevent ethics dumping, safeguard the cultural values and practices of local communities, and simultaneously meet the requirements of individual autonomy and voluntariness of participation. Although there is evidence of potential abuse of power by authoritarian community leaders in highly patriarchal societies, the contribution of gatekeepers in research in African communities cannot be underestimated. Models and frameworks that restrict people's liberty to involve others in their decision-making process could lead to contentions in some African contexts (Appiah, 2021; Behrens, 2018; Ikuenobe, 2018) and needs to be carefully examined. If the overarching goals of research ethics codes and principles are to protect research participants from harm and safeguard their rights of autonomy (Behrens, 2018; Jegede, 2009; Visagie et al., 2019), then the findings of this review seek to suggest that researchers conducting community-based research in Africa should take every measure to ensure that this *right* is also extended to respecting the cultural values of research participants and their communities.

When research engagements are conceived and executed with local population groups (cf. community-based participatory action research; citizen science), then an urgent question is whether IC procedures should be diversified to embrace and respect the cultural values and practices (e.g., social/collective autonomy) of local communities, or to keep assuming that individuals in all contexts make important decisions about their lives on their own. Although the universal ethical codes for research acknowledge that the family and members of the community may be involved in the decision-making process in some settings, there are no specific guidelines that describe in detail the nature of these ethico-cultural and practical challenges and how they can be managed, particularly in the relatively collectivist context of Africa. Given that participatory decision-making is a customary practice in most African settings (Appiah, 2021; Marsh et al., 2019), the ethico-cultural challenges posed by the current individual-focused IC model deserve critical considerations and resolutions.

### Best Practices

The findings of this study can serve as a useful guide to research institutions, researchers, and IRBs in several ways. Firstly, researchers and research institutions can leverage these findings to emphasize the importance of respecting and understanding the local norms, practices, and beliefs of the communities they engage with. This awareness can lead to more culturally sensitive and ethically sound research practices, thereby minimizing ethics dumping and safeguarding the research process. Secondly, IRBs can incorporate the recommendations from the study into their review processes and encourage researchers to adopt a multi-step approach to IC, ensuring that potential participants are adequately informed and have the opportunity to engage with the research process. Thirdly, researchers can work closely with community gatekeepers and leaders, acknowledging their authority and involving them in the research process. This can, in many ways, help build trust and foster mutual collaboration between researchers and local communities. Fourthly, the recommendation urging researchers to conduct a rapid ethical assessment can become a standard practice before initiating research projects in these settings, especially for studies aiming to enroll a large number of participants. This assessment can help identify potential ethical challenges and ways to mitigate them proactively. Lastly, based on the recommendations in the literature, researchers, research institutions, and ethics committees could collaborate to generate and evaluate pluriversal, African-centered IC models that align well with the cultural values and practices of the local people, while also upholding the basic ethical principles of IC.

### Research Agenda

The findings of this review can inform future research agendas by highlighting the context-specific nature of the ethical and cultural challenges associated with the individual-based IC model when implemented in the relatively collectivistic African communities. For instance, researchers can commission studies specific to their particular African contexts to delve deeper into the challenges identified in this scoping review, thereby allowing for a nuanced understanding of how these challenges manifest in different regions and communities. Moreover, comparative studies could be conducted to explore variations in ethico-cultural challenges related to the IC process, identifying both commonalities and differences across diverse African settings, with a concentrated focus on their implications for IC practices. As suggested in the recommendations of the study, there would be need for researchers to engage in collaborative efforts with local communities, community leaders, ethicists, and social scientists to co-create context-specific solutions for IC challenges, as a measure to promote inclusivity and to ensure that the solutions are culturally appropriate. Subsequently, there would be need for longitudinal studies to assess the effectiveness of the implemented solutions over time. This would allow for a dynamic understanding of how these solutions evolve in response to changing cultural dynamics, thereby providing valuable insights into the ongoing refinement of IC practices in the African context.

### Educational Implications

The educational implications of these findings are significant, as they can influence how research ethics is taught and practiced in African educational institutions. The findings of this review implicitly call on educational institutions across the continent to consider revising their curricula to incorporate lessons from field experiences and empirical findings regarding the challenges associated with IC in the relatively collectivistic African contexts. Such an effort can help future researchers and ethicists better understand and navigate these issues. As another important implication, the findings can be a useful resource for institutions to provide guidance to research supervisors on how to mentor students and researchers conducting fieldwork in African communities, with critical considerations for ethical and culturally sensitive research practices. Lastly, the findings serve to provide practical information on the ethico-cultural and practical challenges associated with the individual-based, autonomy-driven IC process across the continent, and thus serve as invaluable resource to inform ethics committees within educational institutions to become more aware of these unique challenges within the African contexts. Based on these findings, ethics committees can further examine the challenges and suggested solutions proffered for their specific regional setting and adapt their review processes to address these challenges and provide guidance to researchers accordingly.

## Supplemental Material

sj-docx-1-jre-10.1177_15562646241237669 - Supplemental material for Balancing Ethics and Culture: A Scoping Review of Ethico-Cultural and Implementation Challenges of the Individual-Based Consent Model in African Research Supplemental material, sj-docx-1-jre-10.1177_15562646241237669 for Balancing Ethics and Culture: A Scoping Review of Ethico-Cultural and Implementation Challenges of the Individual-Based Consent Model in African Research by Richard Appiah, Giuseppe Raviola and Benedict Weobong in Journal of Empirical Research on Human Research Ethics
