# Supplementary material for: Balancing Ethics and Culture: A Scoping Review of Ethico-Cultural and Implementation Challenges of the Individual-Based Consent Model in African Research
Source: J Empir Res Hum Res Ethics. 2024 Mar 18;19(3):143–72. doi: 10.1177/15562646241237669 (PMC11298123; doi:10.1177/15562646241237669)
Supplement: sj-docx-1-jre-10.1177_15562646241237669 - Supplemental material for Balancing Ethics and Culture: A Scoping Review of Ethico-Cultural and Implementation Challenges of the Individual-Based Consent Model in African Research [file sj-docx-1-jre-10.1177_15562646241237669.docx]

**Search strings (not for review).**

| *Search domain* | *Search algorithm* |
| --- | --- |
| Topical | “Consent” OR “Informed consent model” OR “Informed consent process” OR “Consent procedures” OR “Consent practices” |
| Disciplinary | “Biomedical research” OR “Medical research” OR “Social science research” |
| Conceptual | “Ethical challenges” OR “Ethical issues” OR “Ethical considerations” |
| Location | “Africa” OR “African countries” OR “sub-Saharan Africa” OR “Algeria” OR “Angola” OR “Benin” OR “Botswana” OR “Burkina Faso” OR “Burundi” OR “Cabo Verde” OR “Cameroon” OR “Central African Republic” OR “Chad” OR “Comoros” OR “Congo” OR “Djibouti” OR “Egypt” OR “Equatorial Guinea” OR “Eritrea” OR “Eswatini” OR “Ethiopia” OR “Gabon” OR “Gambia” OR “Ghana” OR “Guinea” OR “Guinea-Bissau” OR “Ivory Coast” OR “Kenya” OR “Lesotho” OR “Liberia” OR “Libya” OR “Madagascar” OR "Malawi” OR “Mali” OR “Mauritania” OR “Mauritius” OR “Morocco” OR “Mozambique” OR “Namibia” OR “Niger” OR “Nigeria” OR “Rwanda” OR “Sao Tome and Principe” OR “Senegal” OR “Seychelles” OR “Sierra Leone” OR “Somalia” OR “South Africa” OR “South Sudan” OR “Sudan” OR “Tanzania” OR “Togo” OR “Tunisia” OR “Uganda” OR “Zambia” OR “Zimbabwe” |

**eTable 1: Final Search (PubMed)**

This search was conducted on October 21, 2022, and again on July 28, 2023 (limiting to papers published between October 2022 and July 2023)

| ID | Search Terms and Logic | Results |
| --- | --- | --- |
| #1 | “Consent” OR “Informed consent model” OR “Informed consent process” OR “Consent procedures” OR “Consent practices” | 164,183 |
| #2 | “Biomedical research” OR “Medical research” OR “Social science research” | 675,168 |
| #3 | “Ethical challenges” OR “Ethical issues” OR “Ethical considerations” | 22,869 |
| #4 | “Africa” OR “African countries” OR “sub-Saharan Africa” OR “Algeria” OR “Angola” OR “Benin” OR “Botswana” OR “Burkina Faso” OR “Burundi” OR “Cabo Verde” OR “Cameroon” OR “Central African Republic” OR “Chad” OR “Comoros” OR “Congo” OR “Djibouti” OR “Egypt” OR “Equatorial Guinea” OR “Eritrea” OR “Eswatini” OR “Ethiopia” OR “Gabon” OR “Gambia” OR “Ghana” OR “Guinea” OR “Guinea-Bissau” OR “Ivory Coast” OR “Kenya” OR “Lesotho” OR “Liberia” OR “Libya” OR “Madagascar” OR "Malawi” OR “Mali” OR “Mauritania” OR “Mauritius” OR “Morocco” OR “Mozambique” OR “Namibia” OR “Niger” OR “Nigeria” OR “Rwanda” OR “Sao Tome and Principe” OR “Senegal” OR “Seychelles” OR “Sierra Leone” OR “Somalia” OR “South Africa” OR “South Sudan” OR “Sudan” OR “Tanzania” OR “Togo” OR “Tunisia” OR “Uganda” OR “Zambia” OR “Zimbabwe” | 924,326 |
| #5 | #1 AND #2 AND #3 AND #4 | 88 |
|  | Apply filters: Full text, English, from 2000 – 2023:  (("Consent"[All Fields] OR "Informed consent model"[All Fields] OR "Informed consent process"[All Fields] OR "Consent procedures"[All Fields] OR "Consent practices"[All Fields]) AND ("Biomedical research"[All Fields] OR "Medical research"[All Fields] OR "Social science research"[All Fields]) AND ("Ethical challenges"[All Fields] OR "Ethical issues"[All Fields] OR "Ethical considerations"[All Fields]) AND ("Africa"[All Fields] OR "African countries"[All Fields] OR "sub-Saharan Africa"[All Fields] OR "Algeria"[All Fields] OR "Angola"[All Fields] OR "Benin"[All Fields] OR "Botswana"[All Fields] OR "Burkina Faso"[All Fields] OR "Burundi"[All Fields] OR "Cabo Verde"[All Fields] OR "Cameroon"[All Fields] OR "Central African Republic"[All Fields] OR "Chad"[All Fields] OR "Comoros"[All Fields] OR "Congo"[All Fields] OR "Djibouti"[All Fields] OR "Egypt"[All Fields] OR "Equatorial Guinea"[All Fields] OR "Eritrea"[All Fields] OR "Eswatini"[All Fields] OR "Ethiopia"[All Fields] OR "Gabon"[All Fields] OR "Gambia"[All Fields] OR "Ghana"[All Fields] OR "Guinea"[All Fields] OR "Guinea-Bissau"[All Fields] OR "Ivory Coast"[All Fields] OR "Kenya"[All Fields] OR "Lesotho"[All Fields] OR "Liberia"[All Fields] OR "Libya"[All Fields] OR "Madagascar"[All Fields] OR "Malawi"[All Fields] OR "Mali"[All Fields] OR "Mauritania"[All Fields] OR "Mauritius"[All Fields] OR "Morocco"[All Fields] OR "Mozambique"[All Fields] OR "Namibia"[All Fields] OR "Niger"[All Fields] OR "Nigeria"[All Fields] OR "Rwanda"[All Fields] OR "Sao Tome and Principe"[All Fields] OR "Senegal"[All Fields] OR "Seychelles"[All Fields] OR "Sierra Leone"[All Fields] OR "Somalia"[All Fields] OR "South Africa"[All Fields] OR "South Sudan"[All Fields] OR "Sudan"[All Fields] OR "Tanzania"[All Fields] OR "Togo"[All Fields] OR "Tunisia"[All Fields] OR "Uganda"[All Fields] OR "Zambia"[All Fields] OR "Zimbabwe"[All Fields])) AND ((fft[Filter]) AND (english[Filter])) | 80 |
